# Degradation of an ultrasonically welded device for surgical suture holding

**DOI:** 10.1177/09544119251405597

**Published:** 2025-12-14

**Authors:** Joseph P. Crolla, Elliott Farrell, Lauren E. J. Thomas-Seale, Justin Beyers, Manoj Ramachandran, Martyn Snow, Simon D. Mifsud, Duncan E. T. Shepherd

**Affiliations:** 1 Department of Mechanical Engineering, School of Engineering, University of Birmingham, Birmingham, UK; 2 OsteoWeld Surgical Ltd, Worcestershire, UK; 3 Bonutti Technologies, Effingham, IL, USA; 4 The Royal London Hospital, Barts Health NHS Trust, London, UK; 5 The Royal Orthopaedic Hospital NHS Foundation Trust, Birmingham, UK

**Keywords:** degradation, mechanical testing, PLDLA, PLGA, PLLA, suture, ultrasonic welding

## Abstract

A biodegradable ultrasonically welded device has for the first time been developed for in-body sutures that eliminates the need for surgical knotting. The device comprises two parts that fit together, with a suture inserted between them. Ultrasonic welding is then used to secure the suture by welding the two parts together. The device was manufactured from three biodegradable polymers: Poly(L-lactide-co-D,L-lactide) [PLDLA]; Poly(L-lactide-co-glycolide) [PLGA]; Poly(L-lactide) [PLLA]. All devices were degraded through immersion in phosphate buffer solution at a temperature of 37°C ± 2°C. Knotted sutures on their own were also subject to degradation testing. The devices and knotted sutures were mechanically tested at week zero and after 1, 3 and 6 weeks of degradation. Mechanically testing was undertaken to measure the pull-out strength of sutures from the device. PLGA is not suitable for the device, where a significant reduction in failure force was seen after 3 weeks of degradation. By week 6 the mean failure force (±SD) for PLGA was 74.9 ± 23.4 N, which was significantly less than the use of a suture knot on its own, with a mean failure force of 153.2 ± 37.2 N. PLDLA and PLLA were found to be promising materials, with only a small reduction in mean failure force after 6 weeks of degradation. At week 6 there was no significant difference between the mean failure force of PLDLA, PLLA or the suture knot, with mean failure forces of 152.6 ± 15.0, 128.8 ± 35.0 and 153.2 ± 37.2 N, respectively.

## Introduction

The complete or partial detachment of tendons from their associated bones within the body are common injuries. For example, rotator cuff tears occur in the shoulder when tendons detach from the humeral head. In cases of complete detachment, surgery may be required to reattach the soft tissue to the bone arthroscopically. Numerous devices are currently available to reattach soft tissue to bone with the most common ones using suture anchors. These anchors are placed into the bone with the suture attached.^1^ Traditional knotted suture anchors rely on knot security and surgical knotting arthroscopically is a difficult skill to master. It is often performed inconsistently and sometimes results in slipped knots with suboptimal tension.^2^ Further, knotted sutures can lead to tissue necrosis.^3^

Knotless suture anchors eliminate the need for knots and some studies have found that patients treated with these knotless anchors experience reduced postoperative complications.^4^ However, the sutures tend slip from the anchor at a much lower force (mean force between 66 and 109 N) compared to the force required to pull the anchor from the bone (mean force between 156 and 269 N).^5^

**Figure 1.**
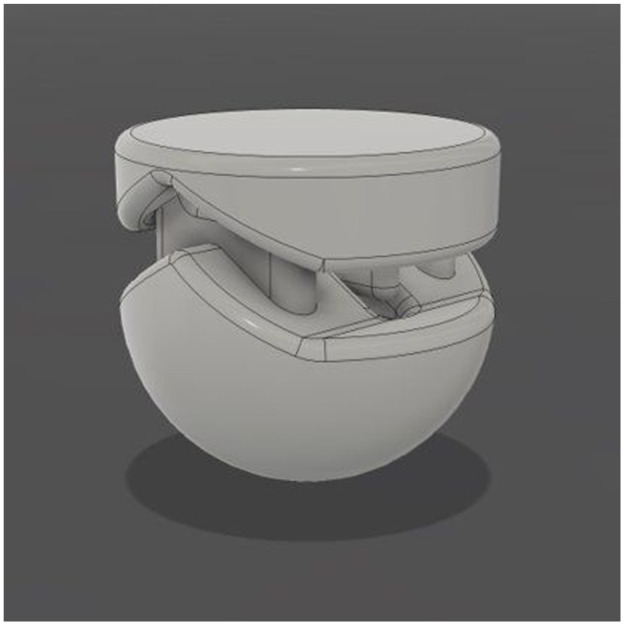
The novel biodegradable ultrasonically welded device for in-body sutures. The two parts of the device fitted together; the diameter of the device is 6 mm. A suture is then inserted through the device and secured using ultrasonic welding.

A novel biodegradable ultrasonically welded device (SutureWeld) has been developed for in-body sutures that eliminated the need for surgical knotting (OsteoWeld Surgical Ltd, Worcestershire, UK). Once developed, SutureWeld would be the first commercially available device of its kind. The device consists of two small polymer parts (Figure 1) that fit together. A suture is then passed through suture channels in the device and the two polymer parts are then ultrasonically welded together to secure sutures during soft tissue surgery, instead of using traditional knots.

Ultrasonic welding uses high frequency vibratory energy to heat and melt the polymer parts together.^6,7^ When the vibrations stop, the molten polymer solidifies, welding the two parts together. Ultrasonic welding has been previously used for various biomaterials such as polyetheretherketone for dental applications,^8^ polyetheretherketone composite for use in an acetabular cup prosthesis,^9^ resorbable poly-(D,L)-lactide (PDLA) copolymer in the reconstruction of the mandibular defects^10^ and the manufacture of biodegradable antibiotic-capsules for drug release.^11^

This is the first study to investigate the use of biodegradable polymers for the device. The aim of this study was to identify a suitable biodegradable polymer for manufacture of the device for surgical suture holding. The aim was achieved by identifying a range of biodegradable polymers that were suitable for ultrasonic welding and measuring the failure force of the sutures from the device at different stages of degradation.

## Materials and methods

### Device manufacture

The biodegradable polymers Poly(L-lactide-co-D,L-lactide) [PLDLA], Poly(L-lactide-co-glycolide) [PLGA] and Poly(L-lactide) [PLLA] were acquired from Evonik Industries (Essen, Germany), with details provided in Table 1. Devices were manufactured from each of the three polymers by Medical Moulded Products (Burton-on-Trent, UK) using injection moulding. To minimise the effect of the hygroscopic behaviour on welding, devices were stored in airtight containers with a desiccant, to maintain a low relative humidity. The devices were sterilised by Steris (Bradford, UK) using gamma radiation at a radiation dose of 31.7–32.9 kGy using a Cobalt-60 source. A total of 144 devices were manufactured for testing, with 48 devices manufactured from each of the three polymers.

**Table 1.** Details of the polymers used in this study.

Material	Material abbreviation	Molar ratio	Inherent viscosity (dL/g)	Product code (Evonik Industries, Essen, Germany)
Poly(L-lactide-co-glycolide)	PLGA	85:15	2.5-3.5	LG 855 S
		L-lactide: glycolide		
Poly(L-lactide-co-D,L-lactide)	PLDLA	70:30	3.3-4.2	LR 706 S
		L-lactide: D,L-lactide		
Poly(L-lactide)	PLLA	N/A	3.3-4.0	L 210 S

### Implant welding

**Figure 2.**
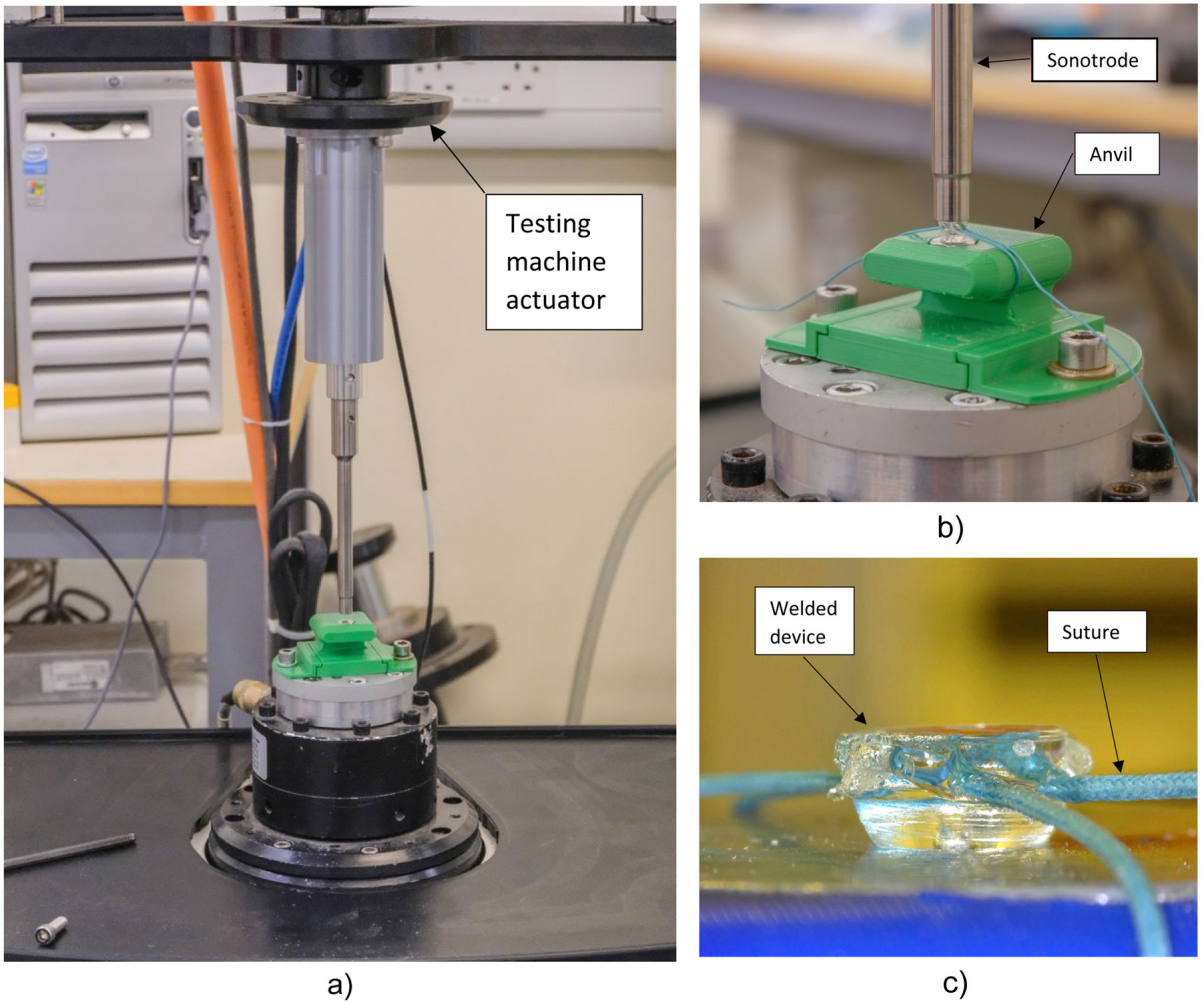
(a) The test set-up used to apply a force of 30 N during welding, (b) the device loaded with a suture on a 3D printed anvil, and (c) the welded device with a suture in place.

The two parts of each device were pressed together and a braided polyester Ethibond suture (USP size 2-0, Ethicon, New Brunswick, New Jersey, USA) was passed through both suture channels, forming a loop. The loop was tightened around a 3D printed anvil (Figure 2), to produce a constant suture loop length of 80 mm. The ultrasonic handpiece was fixed to the actuator of an Electroforce 3300 materials testing machine (TA instruments, New Castle, Delaware, US). The anvil was placed underneath the sonotrode, ensuring the implant was axially aligned with the sonotrode and a compressive force of 30 N was applied to the device. An ultrasonic generator from Sonic Systems (Ilminster, Somerset, UK) was used to apply acoustic energy for 1.25 s, at a frequency of 40 kHz and an amplitude of 5 μm, ultrasonically welding the two parts of the device together and thus securing the suture in place.

### Degradation testing

The standard ASTM F1635 – 95 was used to guide the degradation testing.^12^ All devices were placed in containers and immersed in phosphate buffer solution (Merck, Darmstadt, Germany) with a concentration of 0.01 M. The containers were then placed in a water bath maintained at a temperature of 37°C ± 2°C. The pH of the buffer solution was tested weekly using a pH metre and the solution was replaced if the pH was outside the range of 7.4 ± 0.2. In addition to the polymer devices, knotted sutures on their own were also placed in the water bath.

### Mechanical testing

**Figure 3.**
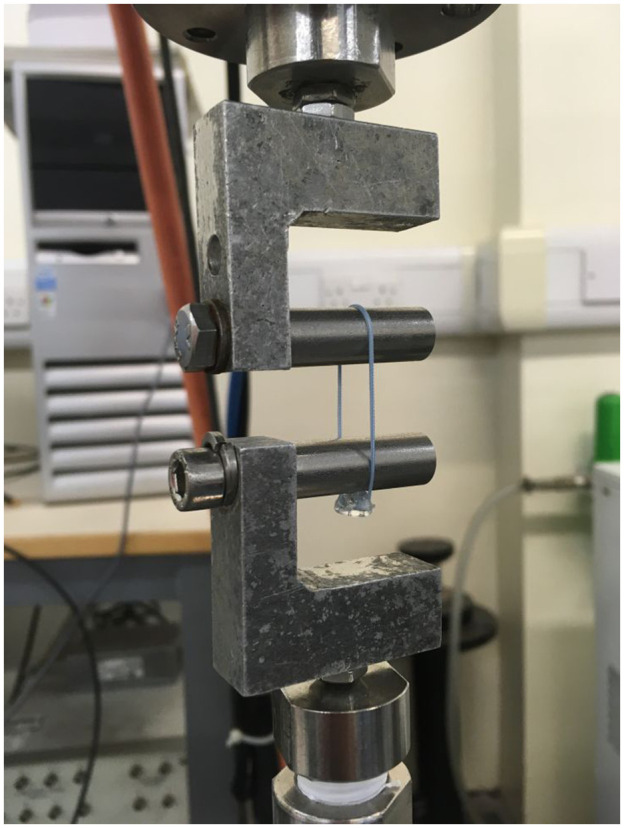
The mechanical test rig showing a suture loop and welded device in position.

Twelve devices made from each of the three polymers devices, along with 12 knotted sutures, were mechanically tested at week zero, before any degradation had taken place. The mechanical testing was undertaken using an Electroforce 3300 materials testing machine. All suture loops were placed around two aluminium cylinders of diameter 12 mm (Figure 3). The upper fixature was attached to the actuator of the testing machine, while the lower fixture was attached to the load cell. The testing machine actuator was set to move at a displacement rate of 1.25 mm/s to place the suture loops into tension. Testing continued until failure, with the failure force and failure mode recorded for all devices. An additional 12 devices of each material and 12 knotted sutures were removed from the water bath after 1, 3 and 6 weeks and were then mechanically tested.

The statistical analysis of the mechanical testing data was performed using Sigmaplot Version 14.5 (Systat Software Inc., San Jose, California, USA). A one-way analysis of variance (ANOVA) plus Tukey pair-wise multiple comparisons was used to compare the results of the mean failure force between all three device materials and the suture knot on its own at week 0 (one way ANOVA) and week 6 (one way ANOVA on ranks). The significance level was set at *p* < 0.05.

## Results

**Figure 4.**
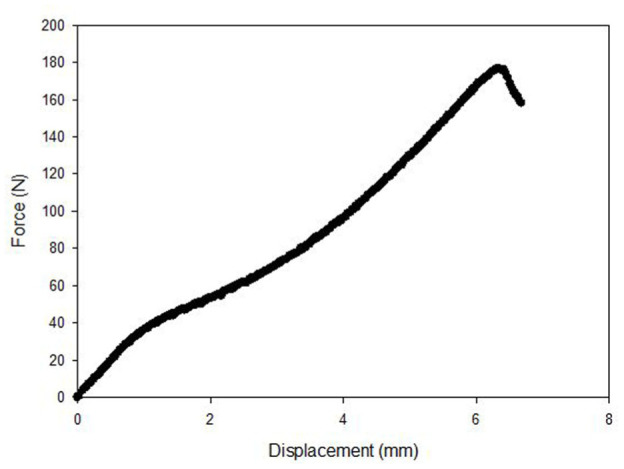
A typical force-displacement curve for a suture pulling out from a PLLA device at week 0.

At week 0 the failure mode for the devices made from PLGA, PLDLA and PLLA was the suture pulling out from the device. Figure 4 shows a typical force-displacement curve for a suture pulling out from a PLLA device at week 0. The failure modes at week 6 were generally different to week 0. For PLGA there was one suture pull-out and 11 device failures. For PLDA there were 10 suture pull outs and two device failures. For PLLA there were five suture pull-outs and seven device failures. At week 0 and week 6 the failure mode for the suture knot on its own was slippage of the knot.

The failure forces for the suture loops are summarised in Table 2. At week 0 the mean failure forces for PLGA, PLDLA and PLLA were in the range 162.7–178.2 N, with no significant difference between the failure force of the three device polymers. At week 0, the mean failure force for the suture knot on its own was 151.1 N, which was significantly lower (*p* < 0.05) than PLDLA and PLLA, but not significantly different from PLGA.

**Table 2.** The descriptive statistics (mean, standard deviation, median, minimum, maximum) of the failure force for suture loops secured with PLLA, PLDLA and PLGA devices, or a suture knot on its own after 0, 1, 3 and 6 weeks of degradation. Each material had 12 devices tested for each week.

Material	Week	Failure force (*N*)				
		Mean	SD	Median	Min	Max
PLGA	0	162.7	24.2	157.0	116	198
	1	125.9	24.4	132.5	90	153
	3	78.3	20.8	70.5	57	120
	6	74.9	23.4	73.0	46	136
PLDLA	0	178.2	18.1	181.5	139	196
	1	158.3	15.6	163.0	126	179
	3	155.0	32.7	162.0	63	185
	6	152.6	15.0	155.0	133	182
PLLA	0	173.7	18.8	174.5	138	208
	1	159.6	29.8	170.0	99	196
	3	153.1	26.4	159.0	113	182
	6	128.8	35.0	132.0	40	179
Suture knot	0	151.2	17.8	148.5	112	175
	1	157.4	18.0	164.5	120	175
	3	164.9	24.0	170.0	124	197
	6	153.2	37.2	164.0	59	192

**Figure 5.**
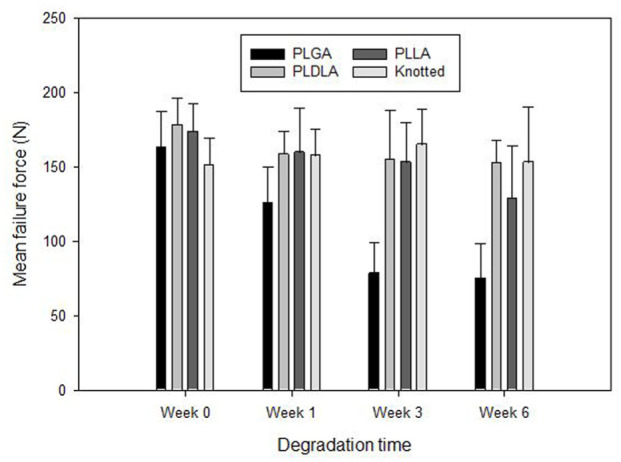
Bar chart showing the mean failure force against degradation time for the PLGA, PLDLA and PLLA devices and a suture knot on its own. The error bars indicate the standard deviations.

The changes in mean failure force for the three device materials from week 0 to week 6 can be seen in Figure 5. PLGA, PLDLA and PLLA all showed reductions in mean failure force from week 0 to week 6. At week 0 the mean failure force for the suture knot was significantly lower than the devices manufactured from PLDLA and PLLA. By week 6, the mean failure force for the PLDLA device, PLLA device and the suture knot were 152.6, 128.8 and 153.2 N, respectively, with no significant difference between the values. For the PLGA device the mean failure force at week 6 was 74.9 N and this was significantly lower (*p* < 0.05) than PLDLA device and the suture knot.

## Discussion

This study investigated the degradation of a novel ultrasonically welded device for surgical suture holding, designed to replace the need for knotted surgical sutures in the repair of soft tissues. The results indicate that PLGA, with an inherent viscosity in the range 2.5–3.5 dL/g, is not a suitable material for the device, where a reduction in mean failure force was observed after 3 weeks of degradation, with a decrease from 162.7 N in week 0 to 78.3 N in week 3. By week 6, the PLGA mean failure force was 74.9 N, which was significantly lower than the mean failure force of 153.2 N for a suture knot on its own at week 6. For the PLGA, the mean failure force reduced more than the other materials possibly due to a faster rate of degradation, but this would require further investigation.

PLDLA and PLLA were found to be more promising material options for manufacturing the device, with both materials showing only a small decrease in mean failure force after 6 weeks of degradation. At week 6, there was no significant difference between the mean failure force of PLDLA, PLLA or the suture knot on its own, with mean failure forces of 152.6, 128.8 and 153.2 N, respectively. While this study has demonstrated that PLDLA and PLLA are suitable materials for the device in principle, further investigation is needed for PLGA, PLDLA and PLLA with higher inherent viscosities. Additionally, the mechanical testing conducted so far has been quasi-static and a detailed understanding of the device under cyclic loading is required.

The results from this study, in terms of failure force for a suture knot on its own and ultrasonically welded materials, are similar to other studies. Richmond^13^ compared loops of polypropylene suture that had been formed using ultrasonic welding with loops formed with traditional knots. The mean failure load for the welded loop was 135.4 N, while the knotted loops failed at 161.9 N. Nho et al.^14^ used a rabbit rotator cuff repair model to compare traditional knots with nylon sutures that had been ultrasonically welded. The mean failure force of the knotted (161.9 N) and welded (161.6 N) groups were similar.

There is a good understanding of the degradation of biodegradable polymers in vitro and in vivo,^15–17^ however, there is little understanding of the effect of ultrasonic welding on the degradation of biodegradable polymers. Sikorska et al.^18^ observed that the welds of PLA (polylactide) packaging started to degrade at a faster rated compared with unwelded areas. Therefore, further investigation is required for the current device.

## Conclusions

This study investigated the degradation of a novel ultrasonically welded device for surgical suture holding. PLDLA and PLLA were found to be promising material options for the device, with both materials only showing a small decrease in mean failure force after 6 weeks of degradation. At week 6 there was no significant difference between the mean failure force of PLDLA, PLLA or the suture knot on its own.
